# Epidemiologic Clues to SARS Origin in China

**DOI:** 10.3201/eid1006.030852

**Published:** 2004-06

**Authors:** Rui-Heng Xu, Jian-Feng He, Meirion R. Evans, Guo-Wen Peng, Hume E Field, De-Wen Yu, Chin-Kei Lee, Hui-Min Luo, Wei-Sheng Lin, Peng Lin, Ling-Hui Li, Wen-Jia Liang, Jin-Yan Lin, Alan Schnur

**Affiliations:** * Guangdong Province Center for Disease Control and Prevention, Guangzhou, China;; † University of Wales College of Medicine, Cardiff, United Kingdom;; ‡ National Public Health Service for Wales, Cardiff, United Kingdom;; § Animal Research Institute, Brisbane, Australia;; ¶ Australian National University, Canberra, Australia;; # World Health Organization, Beijing, China

**Keywords:** SARS, pneumonia, community-acquired infections, disease outbreaks, zoonoses, China, communicable diseases, emerging, child, aged

## Abstract

An epidemic of severe acute respiratory syndrome (SARS) began in Foshan municipality, Guangdong Province, China, in November 2002. We studied SARS case reports through April 30, 2003, including data from case investigations and a case series analysis of index cases. A total of 1,454 clinically confirmed cases (and 55 deaths) occurred; the epidemic peak was in the first week of February 2003. Healthcare workers accounted for 24% of cases. Clinical signs and symptoms differed between children (<18 years) and older persons (>65 years). Several observations support the hypothesis of a wild animal origin for SARS. Cases apparently occurred independently in at least five different municipalities; early case-patients were more likely than later patients to report living near a produce market (odds ratio undefined; lower 95% confidence interval 2.39) but not near a farm; and 9 (39%) of 23 early patients, including 6 who lived or worked in Foshan, were food handlers with probable animal contact.

On March 12, 2003, the World Health Organization (WHO) issued a global alert about cases of atypical pneumonia in Guangdong Province and Hong Kong Special Administrative Region, China, and in Vietnam (*1*). The disease, now known as severe acute respiratory syndrome (SARS), is caused by coronavirus infection (*2**,**3*) and subsequently spread rapidly worldwide. The earliest identified cases of the disease occurred in Guangdong Province in late 2002 (*4*).

On January 2, 2003, two cases of atypical pneumonia in the city of Heyuan, Guangdong Province, were associated with transmission of infection to several healthcare workers at the hospital (*5*). Investigation by the Guangdong Provincial Center for Disease Control and Prevention led to the identification of clusters of cases in six other municipalities (Foshan, Jiangmen, Zhongshan, Guangzhou, Shenzhen, Zhaoqing) from November 2002 to mid-January 2003. On February 3, 2003, province-wide mandatory case reporting of atypical pneumonia that used a standard case definition and reporting form was instituted. The provincial health department also introduced a range of public health control measures, including guidelines on epidemiologic investigation of cases and contacts (February 3) and on hospital admission, clinical management, and infection control arrangements for patients (February 9). Subsequently, the department issued guidelines on community prevention and control, including mandatory home quarantine of contacts (March 27); commenced public service announcements about personal protection and seeking prompt medical attention (March 27); and introduced free hospital treatment for patients with SARS (April 30). Border control measures were introduced at all points of entry into the province during mid-April according to WHO recommendation (*6*). We describe the epidemiology of the SARS epidemic in Guangdong through April 30, 2003, focusing on the observed pattern of spread of disease, the signs and symptoms, and the investigation of early cases.

## Methods

### Study Population

Guangdong Province has a population of 85.2 million, including 9.9 million in Guangzhou city (*7*). All public health and most hospital services are under the direction of the Health Bureau of Guangdong Provincial People’s Government. The public health function is performed by one provincial Center for Disease Control and several municipal centers, together with a network of district and county centers, each responsible for a population of 500,000–1 million. Nearly all hospitals are operated by the public sector, but patients are charged for medical treatment. Primary health care in the province is rudimentary, and most patients report directly to hospital emergency rooms.

### Data Sources

We analyzed data from two sources: the Guangdong surveillance database and a case investigations database. We also interviewed staff from the Guangdong Provincial Centers for Disease Control, and Foshan Municipal Center for Disease Control to obtain supplementary information on early-onset cases. Information on early cases in the neighboring Guangxi Province was obtained from local investigators by a visiting WHO team, led by one of the authors (CKL). Early cases were defined as those with a date of onset from November 1, 2002, to January 31, 2003, and late cases as those with a date of onset from February 1 to April 30, 2003. Population denominators were obtained from the Guangdong provincial census for 2000 (*7*).

### Surveillance Database

Guangdong Provincial Center for Disease Control coordinated the surveillance database. Early cases were identified during the course of case investigations or after voluntary reporting by clinicians. Such cases were only included in the database if they conformed to the case definition subsequently adopted for surveillance. Since early February 2003, hospitals at all levels in the health system were required to report cases of atypical pneumonia (probable SARS) immediately by telephone to the local center for disease control, which then forwarded reports electronically to the provincial center on the same day. The diagnostic criteria for reporting were: 1) having close contact with a patient or having infected other people, 2) fever (>38°C) and symptoms of respiratory illness, 3) leukocyte count <10.0 x 10^9^/L, 4) radiographic evidence of infiltrates consistent with pneumonia or respiratory distress syndrome on chest x-ray, and 5) no response to antimicrobial drug treatment (within 72 hours). Patients were considered to be probable SARS patients if they meet criteria 1–4 or 2–5 but were excluded if an alternative diagnosis could fully explain their illness. The dataset contains patient demographics, including occupation; dates of onset, admission and report; criteria required for the case definition; and details of laboratory specimens that were collected.

### Case Investigation Database

Contact tracing staff at the district center level administered a standard questionnaire to all case-patients within 24 hours of reporting. These data form the basis of the case investigation database and comprise patient demographics; clinical features on admission to hospital; contact history (living with, working with, caring for, or visiting the home of a patient) and name, age, and address details of contacts; and exposure risk factors for community cases (non-healthcare workers), including travel history, hospital visits, animal contact, and living conditions. Patients not employed as healthcare workers were classified as community case-patients. Comparisons were made between features of early-onset and late-onset community cases, and primary (no contact history) and secondary community cases. Extra information was collected on early patients by means of informal interviews with center staff, which focused on index patients in each of the seven municipalities initially affected. Data were particularly sought on occupational history and contact networks, and a detailed case series was compiled.

### Data Analysis

Data were entered into Excel databases (Microsoft, Redmond, WA). Descriptive analyses were carried out using EpiInfo version 6 (Centers for Disease Control, Atlanta, GA) and SPSS version 10.0 (SPSS Inc., Chicago, IL). The surveillance database was used for analysis by age, sex, occupation, and clinical signs and symptoms. The case investigation database was used for comparing early- and late-onset cases and cases with or without a contact history. Incidence rates were calculated for November 2002 to April 2003. For comparisons of signs and symptoms by age, younger adults were used as the reference group for both children and older persons. Chi-square test or, when appropriate, Fisher exact test was used for comparison of proportions, Mann-Whitney test for comparison of continuous variables, and chi-square test for linear trend for analysis of time trends. We report maximum likelihood estimates for odds ratios (OR) with exact 95% mid-p confidence intervals (CI) and consider p < 0.05 to be significant.

## Results

### Surveillance Database

A total of 1,454 SARS cases were reported in Guangdong Province from November 16, 2002, through April 30, 2003, including 55 deaths, a crude case-fatality rate of 3.8% for all ages, and 12.7% in people >65 years. Two children died: a 4-year-old, previously healthy girl with lobar pneumonia of unknown cause and a 10-year-old boy with recent acute hepatitis B.

**Figure 1:**
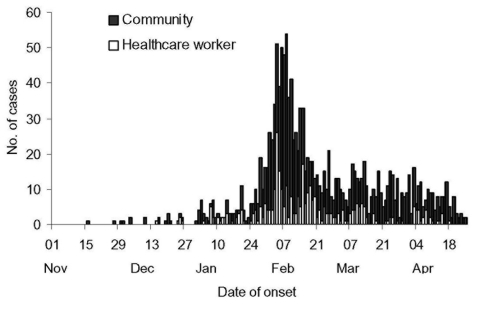
Epidemic curve of cases of severe acute respiratory syndrome by date of onset, November 1, 2002–April 30, 2003, in Guangdong Province, China, showing cases in the community and in healthcare workers.

**Figure 2:**
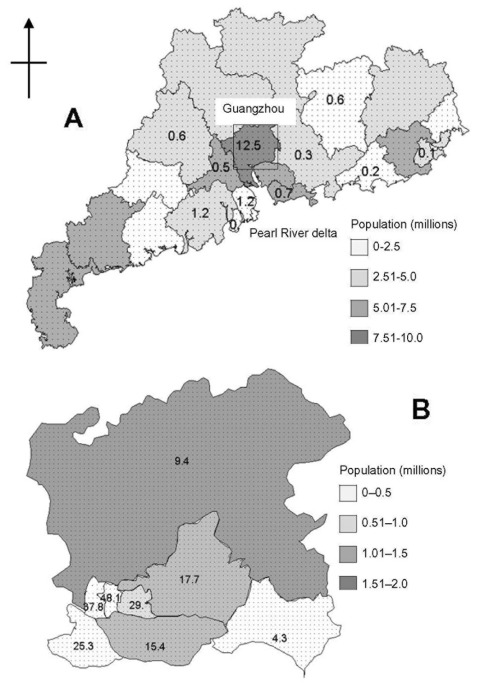
Geographic distribution of population in: (A) urban districts of Guangzhou city, (B) Guangdong Province and district-specific incidence of severe acute respiratory syndrome (per 100,000 population).

**Figure 3:**
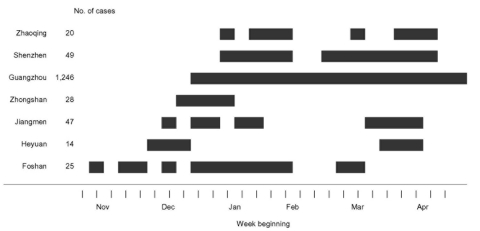
Timeline of cases of severe acute respiratory syndrome by week of onset, November 1, 2002–April 30, 2003, in the seven predominantly affected municipalities of Guangdong Province, China.

The initial phase of the epidemic, November–late January, was characterized by sporadic cases followed by a sharp rise in late January and a sharp decline in the first half of February, and thereafter a gradual decline (Figure 1). The epidemic peak occurred at the end of the first week of February with approximately 55 new cases each day. Cases occurred in 15 municipalities in the province but were concentrated in the Pearl River delta area (Figure 2A), and confined almost exclusively to urban areas, particularly the seven municipalities of Foshan, Guangzhou, Heyuan, Jiangmen, Shenzhen, Zhongshan, and Zhaoqing (located 20–130 km from Guangzhou). The highest incidence occurred in Guangzhou city (12.5 cases per 100,000 people) (Figure 2B), and outbreaks appear to have occurred in different municipalities at varying stages of the epidemic (Figure 3).

**Figure 4:**
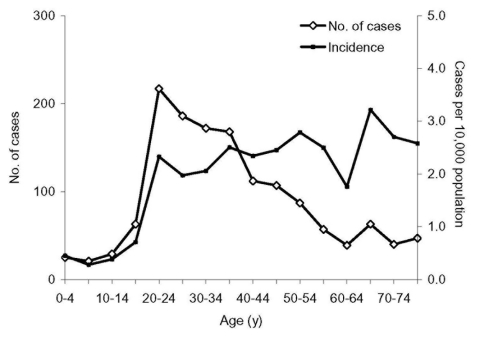
Number of patients with severe acute respiratory syndrome by age, and age-specific incidence (per 10,000 population), November 1, 2002–April 30, 2003, Guangdong Province, China.

Median age of patients was 35.0 years (range 0–92 years), and the highest age-specific incidence was in persons 65–69 years (3.2 per 100,000 people) (Figure 4); 53.2% of cases were female. Five deaths occurred among 343 cases in healthcare workers (24% of 1,429 cases for whom occupation is known); 75.1% of healthcare worker cases were in women. The proportion of healthcare worker patients was highest in the early phase of the epidemic (32% with date of onset in January 2003) and declined as the epidemic progressed (27% in February, 18% in March, and 17% in April) (Table 1). Throughout the epidemic, a high proportion of community case-patients did not report contact history, ranging from 58% in February to 74% in April. This proportion was even higher in children (91% in those <5 years, 81% in those 5–14 years) and in persons >65 years (82%).

**Table 1:** Month of onset of SARS in community case-patients and in healthcare workers, Guangdong, China, November 2002–April 2003^a^

Month of onset	Community case-patient contact history			
	Yes (%)	No (%)	Healthcare worker (%)	Total^b^
Nov 2002	0 (0)	3 (100)	0 (0)	3 (100)
Dec 2002	2 (11)	12 (63)	5 (26)	19 (100)
Jan 2003	31 (18)	87 (50)	55 (32)	173 (100)
Feb 2003	104 (14)	419 (58)	195 (27)	718 (100)
Mar 2003	63 (20)	197 (62)	59 (18)	319 (100)
Apr 2003	16 (9)	129 (74)	29 (17)	174 (100)
Total	216 (15)	847 (60)	343 (24)	1,406 (100)

^a^SARS, severe acute respiratory syndrome. ^b^Information was not available on contact history for 48 cases.

Analysis of occupation status, excluding healthcare workers and case-patients with known exposure, shows that the proportion of cases in students (0% in January 2003 or before; 7% in February; 14% in March; 18% in April, p < 0.001) and housewives (0% in January 2003 or before; 5% in February; 14% in March; 15% in April, p < 0.001) increased as the epidemic progressed (Table 2). A high proportion (9/23, 39%) of early cases were food handlers (this category includes persons who handle, kill, and sell food animals, as well as those who prepare and serve food), but none were farmers handling livestock or poultry. Of the nine early cases in food handlers, seven were restaurant chefs working in township restaurants (where a variety of animals were slaughtered on the premises), one was a market produce buyer for a restaurant, and one was a snake seller in a produce market (where a variety of live animals were offered for sale). Six of the food handlers lived or worked in Shunde (1.7 million population), an urban district of Foshan municipality, though none could be directly linked to each other through contact history.

**Table 2:** SARS cases (%) by month of onset and occupational status, Guangdong, China, November 2002–April 2003^a^

Occupational status^b^	Jan 2003 or before no. (%)	Feb 2003 (%)	Mar 2003 (%)	Apr 2003 (%)	Total (%)
Retired	2 (9)	44 (10)	46 (23)	32 (16)	124 (15)
Worker	2 (9)	40 (9)	28 (14)	22 (11)	92 (11)
Student	0 (0)	29 (7)	28 (14)	34 (18)	91 (11)
Civil servant	3 (13)	43 (10)	26 (13)	19 (10)	91 (11)
Housewife	0 (0)	20 (5)	28 (14)	30 (15)	78 (9)
Food industry worker	9 (39)	20 (5)	4 (2)	19 (10)	52 (6)
Farmer	1 (4)	10 (2)	4 (2)	4 (2)	19 (2)
Teacher	1 (4)	7 (2)	6 (3)	4 (2)	18 (2)
Child	0 (0)	9 (2)	4 (2)	4 (2)	17 (2)
Other	2 (9)	49 (11)	14 (7)	18 (9)	83 (10)
Unknown	3 (13)	157 (37)	14 (7)	8 (4)	182 (21)
Total	23 (100)	428 (100)	202 (100)	194 (100)	847 (100)

^a^SARS, severe acute respiratory syndrome. ^b^Excluding healthcare workers or case-patients with known exposure.

### Case Investigation Database

Detailed data from case investigation interviews were available for 662 (45%) of 1,454 patients. Median age was 31.0 years (range 0–86 years), 56% were female, and 44% were male. The signs and symptoms in adults (18–64 years) were compared with those in children (<18 years) and older persons (>65 years) (Tables 3 and 4). Children were more likely to have a runny nose and dry cough on physical examination but less likely to have chills, malaise, headache, muscle aches, or difficulty breathing. Older persons were more likely to report having sputum and to have a dry or productive cough on physical examination but less likely to complain of chills, malaise, or sore throat. Nearly all patients had a high body temperature (median 39.0°C, range 36.8–42.0°C) lasting in most patients for 1 to 4 days (median 4.0 days; range 1–9 days). Median leukocyte count was 5.8 x 10^9^/L (range 1.0–63.4 x 10^9^/L), and 13.9% of patients had leukopenia (<3.5 x 10^9^/L). Older persons had a higher median leukocyte count than younger adults (6.6 x 10^9^/L for those >65 years, 5.6 x 10^9^/L for those 18–64 years, p = 0.056), and fewer had leukopenia.

**Table 3:** Prevalence (%) of symptoms on admission to hospital, SARS patients, Guangdong, China, November 2002–April 2003^a^

Variable	All (n = 662)	Adults (18–64 y) (n = 534)	Children (<18 y) (n = 51)	OR (95% CI)	p	Older persons (> 65 y) (n = 66)	OR (95% CI)	p
Fever	97.4	97.4	98.0	1.4 (0.2 to 29.3)		98.5	1.8 (0.3 to 37.9)	
Chills	51.8	56.0	31.4	0.4 (0.2 to 0.7)	0.002	37.9	0.5 (0.3 to 0.8)	0.008
Malaise	42.3	45.7	23.5	0.4 (0.2 to 0.7)	0.004	31.8	0.5 (0.3 to 1.0)	
Headache	40.0	42.1	25.5	0.5 (0.2 to 0.9)	0.03	43.1	0.7 (0.4 to 1.3)	
Muscle ache	30.8	35.0	5.9	0.1 (0.0 to 0.3)	<0.001	16.7	0.4 (0.2 to 0.7)	0.004
Cough	69.8	60.0	82.4	2.2 (1.1 to 4.9)	0.05	80.3	1.9 (1.1 to 3.8)	0.05
Sputum	38.2	36.9	39.2	1.7 (0.9 to 3.3)		51.5	1.8 (1.1 to 3.1)	0.03
Sore throat	16.3	16.7	25.5	1.1 (0.6 to 2.0)		6.1	0.3 (0.1 to 0.8)	0.04
Runny nose	7.4	6.7	15.7	2.6 (1.1 to 5.7)	0.04	7.6	1.1 (0.4 to 2.8)	
Breathing difficulty	26.7	27.0	11.8	0.4 (0.1 to 0.8)	0.03	43.3	1.8 (1 to 3.0)	0.05
Nausea	8.8	9.4	5.9	0.6 (0.1 to 1.8)		7.6	0.8 (0.3 to 2.0)	
Vomiting	6.2	6.0	7.8	1.3 (0.4 to 3.7)		7.6	1.3 (0.4 to 3.3)	
Diarrhea	8.6	9.0	7.8	0.9 (0.3 to 2.3)		7.6	0.8 (0.3 to 2.2)	

^a^SARS, severe acute respiratory syndrome; OR, odds ratio, using adults as the reference group; CI, confidence interval.

**Table 4:** Prevalence (%) of physical signs, chest x-ray findings, and blood count abnormalities on admission to hospital, SARS patients, Guangdong, China, November 2002–April 2003^a^

Variable	All (n = 662)	Adults (18–64 y) (n = 534)	Children (<18 y) (n = 51)	OR^b^ (95% CI)	p	Older persons (>65 y) (n = 66)	OR^b^ (95% CI)	p
Physical signs								
Temperature (>38°C)	91.3	90.9	97.8	0.9 (0.3 to 3.9)		89.7	0.5 (0.1 to 1.2)	
Rigors	15.9	16.9	10.9	0.5 (0.2 to 1.3)		10.6	0.6 (0.2 to 1.2)	
Lethargy	10.3	11.8	2.0	0.2 (0.0 to 0.8)		4.5	0.4 (0.1 to 1.1)	
Myalgia	6.6	8.1	2.0	0.2 (0.0 to 1.2)		0.0	0.0 (0.0 to 0.6)	0.009
Cough	50.0	46.6	64.7	2.1 (1.9 to 3.9)	0.02	68.2	2.5 (1.4 to 4.3)	0.002
Sputum	10.4	8.6	13.7	1.7 (0.7 to 3.8)		24.2	3.4 (1.8 to 6.4)	<0.001
Dyspnea	6.0	5.8	2.0	0.3 (0.0 to 1.8)		12.1	2.2 (0.9 to 4.5)	
Clinical test results								
Abnormal chest x-ray	87.2	87.5	84.3	0.8 (0.4 to 1.8)		86.4	0.9 (0.4 to 2.0)	
Leukopenia (<3.5x10^9^/L)	13.9	14.2	16.3	1.2 (0.5 to 2.7)		8.3	0.6 (0.2 to 1.5)	

^a^OR, odds ratio; CI, confidence interval. ^b^Using adults as the reference group.

Comparison of case categories indicate that community case-patients with a contact history were more likely to have visited a hospital in the previous 2 weeks than patients without a contact history (OR 6.83, 95% CI 2.89 to 16.73) (Table 5). Patients without a contact history were no more likely to report a history of travel or animal contact. Early-onset patients were more likely to live within walking distance of a produce market (an agricultural market where live animals are sold, killed, and butchered in situ, also known as a "wet market") than late-onset patients (OR undefined, lower 95% CI 2.39). Living near a poultry or livestock farm or having other types of animal contact, including domestic pets or livestock, poultry, or specific wild animals or birds, was not associated with a high risk for SARS.

**Table 5:** Case-case comparisons of community SARS patients, Guangdong, China, November 2002–April 2003, according to contact history and onset date^a^

Exposure (in previous 2 weeks)	No contact history (n = 406)		Contact history (n = 103 )			Early onset^b^ (n = 19)^c^		Late onset^b^ (n = 387)^c^		
	Yes	No	Yes	No	OR (95% CI)	Yes	No	Yes	No	OR (95% CI)
Visited hospital	17	70	22	13	6.83 (2.89 to 6.73)	0	10	45	169	0.00 (0.00 to 1.36)
Visited by a friend	4	71	1	17	1.04 (0.04 to 8.93)	0	1	4	70	0.00 (0.00 to 337)
Regular hand washing	122	15	44	3	1.80 (0.53 to 8.10)	4	1	118	14	0.48 (0.06 to 12.55)
Travel history	45	179	13	62	0.83 (0.41 to 1.63)	0	10	45	169	0.00 (0.00 to 1.36)
Animal contact	37	262	13	56	1.64 (0.80 to 3.25)	1	3	36	259	2.39 (0.09 to 23.02)
Visited produce market	9	79	1	19	0.41 (0.02 to 2.75)	0	1	9	69	0.00 (0.00 to 148)
Lives near produce market	89	169	19	43	0.84 (0.45 to 1.52)	5	0	84	169	Undef. (2.39 to Undef.)
Lives near poultry or livestock farm	6	252	3	59	2.13 (0.42 to 8.81)	0	19	6	247	0.00 (0.00 to 40.15)

^a^SARS, severe acute respiratory syndrome; CI, confidence interval; OR, odds ratio. ^b^Defined as November 1, 2002–January 31, 2003 for early onset; February 1, 2003–April 30, 2003 for late onset. ^c^Cases with no contact history and for whom case investigation data are available.

### Case Series of Index Patients

The index patients in each of the seven earliest affected municipalities all had a date of onset before January 31, 2003 (Table 6). In five municipalities (Foshan, Jiangmen, Zhongshan, Guangzhou, Shenzhen), outbreaks appear to have occurred independently, but the outbreak in Heyuan may be linked to that in Shenzhen and the outbreak in Zhaoqing to that in Guangzhou. Index patients from the eight other municipalities involved in the epidemic had a date of onset after March 1, 2003, and a travel history to an affected area, so these were excluded from the analysis.

**Table 6:** Case series of index cases by municipality in SARS epidemic, Guangdong, China, November 2002–April 2003^a^

Case no.	City	Sex	Age	Occupation	Date of onset	Animal contact	Secondary transmission
Case 1	Foshan	M	45	Administrator and village leader	Nov 16, 2002	Yes	Yes
Case 2	Heyuan	M	34	Restaurant chef	Dec 10, 2002	Unknown	Yes
Case 3	Jiangmen	M	26	Factory worker	Dec 21, 2002	No	No
Case 4	Zhongshan	M	30	Restaurant chef	Dec 26, 2002	Yes	Yes
Case 5	Guangzhou	M	49	Office worker	Jan 2, 2003	No	Yes
Case 6	Shenzhen	M	46	Office worker	Jan 15, 2003	No	Yes
Case 7	Zhaoqing	F	39	Market vendor	Jan 17, 2003	Probably	Yes

^a^SARS, severe acute respiratory syndrome; M, male; F, female.

**Figure A1:**
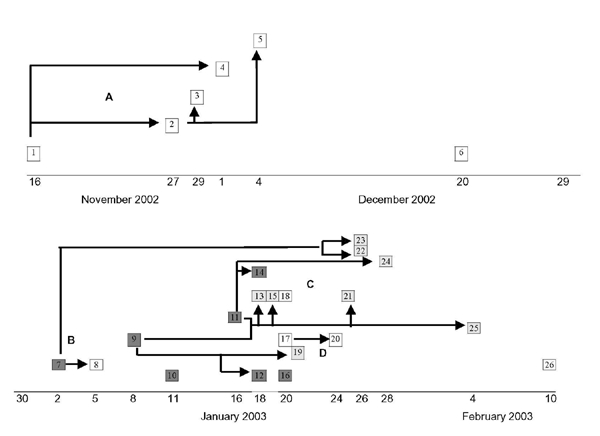
Timeline of cases of severe acute respiratory syndrome by date of onset and chains of transmission, November 16, 2002-February 10, 2003, Foshan municipality, Guangdong Province, China. A: Index patient and family cluster. B: Family and healthcare workers cluster. C: Healthcare worker cluster at township hospital. D: Workplace cluster. Case-patients 6, 10, 18, and 26 had no contact history. Dark box: food handler, light box: healthcare worker.

Patient 1 had the earliest case, identified by retrospective case searching. He lived with his wife and four children in Foshan city and became ill on November 16, 2002. He had not traveled outside Foshan in the 2 weeks before his illness and had no contact history, but he had prepared food including chicken, domestic cat, and snake. He was part of a cluster of five patients, including his wife (42 years old, onset December 1), a 50-year-old aunt who visited him in the hospital on November 20 (onset November 27), and the aunt’s 50-year-old husband (onset November 30) and 22-year-old daughter (onset December 4) (Figure A1, Cluster A). None of patient l’s four children were ill. Subsequent clusters in Foshan included a food handler who infected a family member and two healthcare workers (Figure A1, Cluster B) and a food handler who infected several healthcare workers (Figure A1, Cluster C).

Patient 2 lived in Heyuan but worked as a restaurant chef in Shenzhen. His work was mainly stir-frying and did not involve killing animals. His animal contact history is unknown. He returned to Heyuan after becoming ill, was admitted to the local hospital and transferred to Guangzhou 2 days later. He infected a work colleague (41-year-old man, onset December 16), six healthcare workers in Heyuan (onset December 24–28), and a physician who accompanied him in the ambulance from Heyuan to Guangzhou (28-year-old man, onset December 25). Patient 3, from Jiangmen, had no contact history, no history of animal contact, and no known forward transmission. Patient 4 worked as a chef in a Zhongshan township restaurant, where he prepared steamed dishes and had contact with snakes, civet cats, foxes, and rats. He infected his 30-year-old wife (onset January 3), a 39-year-old male friend who visited him in the hospital (onset January 5), and a physician (35-year-old man, onset January 4). Patient 4 was one of two patients responsible for infecting at least three healthcare workers (onset January 4–7). Patient 5, from Guangzhou, had no history of animal contact other than with a pet guinea pig that died a month before his symptoms began. He infected a hospital intern (onset January 14) and six other healthcare workers (onset January 14–22) at Guangdong Traditional Chinese Medicine Hospital. Patient 6, from Shenzhen, had visited Hong Kong on January 14, the day before symptom onset. However, he had no contact history or contact with animals. He infected a work colleague (43-year-old man, onset January 29) and died 14 days after becoming ill. Patient 7, from Zhaoqing, was the only female index case. She traveled to Guangzhou 2 weeks before becoming ill, although she could not recollect contact with anyone with symptoms of SARS. She worked at a market but did not sell animals. She infected her 16-year-old son (onset February 3) and a physician (25-year-old woman, onset January 31).

The index patient in the neighboring province of Guangxi was a 26-year-old man, who infected several family members. He worked as a driver for a wild animal dealer and returned home to Guangxi after becoming ill on January 8, 2003. The dealer supplied Guangdong markets with wild animals from Guangxi, other Chinese provinces, and Vietnam.

## Discussion

The epidemic of atypical pneumonia in Guangdong Province that we describe bears all the hallmarks of SARS (*8**–**11*). It demonstrates the typical time course of the epidemic, the preponderance of cases in urban areas, and the epidemiologic and clinical features of the disease. Since the SARS epidemic began in Guangdong, we have sought epidemiologic clues about the origin of the disease. Approximately 75% of emerging infectious diseases are zoonotic (*12*), and evidence is accumulating that an animal origin for SARS is probable. However, phylogenetic analysis and sequence comparisons of the coronavirus that causes SARS (SARS-CoV) indicate that the virus is not closely related to any of the previously characterized human or animal coronaviruses (*13*). The reservoir is still unknown, but SARS-CoV has been isolated from Himalayan palm civets (*Paguma larvata*), and evidence of infection has been found in a raccoon dog (*Nyctereutes procyonoides*), a Chinese ferret-badger (*Melogale moschata*), and humans working at a live animal market in Shenzen municipality (*14*). Seroprevalence of immunoglobulin (Ig) G antibody to SARS-CoV is substantially higher among traders of live animals (13.0%) in Guangzhou municipality than among healthy controls (1.2%), and the highest prevalence of antibody is among those who traded primarily masked palm civets (72.7%) (*15*). The pattern of the Guangdong epidemic is consistent with the classical process of emergence from an animal reservoir: the initial introduction of the virus into a nonimmune human population followed by the establishment and rapid dissemination of infection (*16*). The traditional practice of using wildlife for food and medicine, still observed by some persons in southern China, offers an effective bridge from a natural animal host to humans. Several observations support this hypothesis. Two of the seven index patients were restaurant chefs; food handlers (who handle, kill, or butcher animals) were overrepresented among early-onset cases with no contact history (including the first reported death, in a snake seller); and patients with early onset were more likely than patients with late onset to live near an agricultural produce market (where live wild animals are generally offered for sale). However, none of the early patients were commercial farmers nor was living near a farm associated with increased risk, findings that suggest a wild animal rather than a livestock or poultry source. Although trade in wildlife is now illegal in China, a range of mammalian, avian, and reptile species can still be found in some produce markets, and a black market in these species probably exists. Many such animals come from outside Guangdong Province, often through Guangxi Province to the west, and may originate in Vietnam or other parts of Southeast Asia. The observation that the index patient in Guangxi Province was a wild animal trader who supplied Guangdong markets offers some circumstantial evidence for such a link.

Our data have several limitations. First, surveillance for SARS was only established in February 2003; thus, information on earlier cases was collected retrospectively and will be influenced by reporting bias. Second, our analysis is based on cases that are not laboratory confirmed. Thus, the diagnosis relies on a clinical case definition and the sensitivity and specificity are unknown. Third, case investigation data were only available on approximately half of all cases because of poor transfer of data (regardless, all categories of cases and all reporting districts were similarly affected). Finally, information on several earlier cases was incomplete or may be unreliable (because of fear of prosecution associated with the trade in wild animals), and some persons are no longer traceable.

The data also highlight several unanswered questions. The SARS epidemic started in Guangdong, but how it began, why it peaked so suddenly, why Guangzhou was so badly affected and other cities spared, and what caused the gradual decline are all unclear. The decline in the epidemic is probably a result of hospital and community infection control measures introduced in early February, including strict isolation of patients, use of protective equipment by healthcare workers, prohibition of hospital visitors, and guidelines on epidemiologic investigation. Such measures may also explain why later cases did not trigger such extensive chains of transmission. The concentration of cases in urban areas may be due to factors associated with access to healthcare or to incomplete or poor surveillance in rural areas. However, in Guangdong Province, emphasis was placed on reporting from less developed prefectures and rural areas, including supervisory visits and review of hospital records. Many larger cities in Guangdong, as well as rural areas, were also apparently spared by the epidemic.

Although the possibility that SARS may pre-date the earliest known case cannot be excluded, the temporal and spatial clustering of index cases described in the case series suggests that the initial source of the Guangdong epidemic was either a single point source (with the links between cities not identified) or several point sources in the Pearl River basin. Outbreaks in cities affected later in the epidemic can all be traced to an imported case. These later cases are probably due to horizontal transmission rather than to further contact with the initial source. The reason for the sudden rise in the incidence of cases at the beginning of February is unclear, although the rise coincides with the admission of a highly infectious index patient who transmitted SARS to healthcare workers at three different hospitals in Guangzhou city and to a large number of family members (*5**,**17*). The absence of such a trigger may explain the much smaller outbreaks in other cities in the province. The case-fatality rate in Guangdong was also lower than documented elsewhere (*11**,**18*). The most likely explanation for this lower rate is the younger age structure of the population in mainland China compared to that of Hong Kong, Taiwan, or Canada. Children have slightly different initial signs and symptoms than adults perhaps because symptoms are milder (*19*), children are less able to describe their symptoms, or the case definition is less specific in this age group. Older persons are more likely to have a productive cough and difficulty breathing than younger adults, which suggests either a misdiagnosis or an underlying chest disease.

The high proportion of community case-patients with no apparent contact history, especially in Guangzhou city, may be due to inadequate tracing, poor reporting of the results of contact investigation, asymptomatic transmission, or the nonspecificity of the clinical case definition. Similar findings have been observed in Beijing (*20*). Little evidence as yet exists for asymptomatic infection with SARS, but seroprevalence studies will help determine whether this occurs. Laboratory testing of stored clinical specimens may also clarify the specificity of the case definition, particularly if positivity rates vary during different stages of the epidemic or in cases with no contact history. An alternative explanation for the absence of a contact history is the continuing existence of an environmental source; however, this explanation is not borne out by case investigation data. Clarifying this factor is important not only to help understand the transmission dynamics of SARS but also to allay concerns about the risk for epidemic spread in the community if SARS is reintroduced. Resolving this issue will be vital to prospects for preventing the spread of SARS beyond China (*21*).
